# Development and implementation of a radiation therapy incident learning system compatible with local workflow and a national taxonomy

**DOI:** 10.1002/acm2.12218

**Published:** 2017-11-22

**Authors:** Logan Montgomery, Palma Fava, Carolyn R. Freeman, Tarek Hijal, Ciro Maietta, William Parker, John Kildea

**Affiliations:** ^1^ Medical Physics Unit Department of Physics McGill University Montréal Canada; ^2^ Division of Radiation Oncology Department of Oncology McGill University Montréal Canada; ^3^ Medical Physics Unit Department of Oncology McGill University Montréal Canada

**Keywords:** incident, incident learning, incident report, NSIR‐RT, radiation therapy, software

## Abstract

**Purpose:**

Collaborative incident learning initiatives in radiation therapy promise to improve and standardize the quality of care provided by participating institutions. However, the software interfaces provided with such initiatives must accommodate all participants and thus are not optimized for the workflows of individual radiation therapy centers. This article describes the development and implementation of a radiation therapy incident learning system that is optimized for a clinical workflow and uses the taxonomy of the Canadian National System for Incident Reporting – Radiation Treatment (NSIR‐RT).

**Methods:**

The described incident learning system is a novel version of an open‐source software called the Safety and Incident Learning System (SaILS). A needs assessment was conducted prior to development to ensure SaILS (a) was intuitive and efficient (b) met changing staff needs and (c) accommodated revisions to NSIR‐RT. The core functionality of SaILS includes incident reporting, investigations, tracking, and data visualization. Postlaunch modifications of SaILS were informed by discussion and a survey of radiation therapy staff.

**Results:**

There were 240 incidents detected and reported using SaILS in 2016 and the number of incidents per month tended to increase throughout the year. An increase in incident reporting occurred after switching to fully online incident reporting from an initial hybrid paper‐electronic system. Incident templating functionality and a connection with our center's oncology information system were incorporated into the investigation interface to minimize repetitive data entry. A taskable actions feature was also incorporated to document outcomes of incident reports and has since been utilized for 36% of reported incidents.

**Conclusions:**

Use of SaILS and the NSIR‐RT taxonomy has improved the structure of, and staff engagement with, incident learning in our center. Software and workflow modifications informed by staff feedback improved the utility of SaILS and yielded an efficient and transparent solution to categorize incidents with the NSIR‐RT taxonomy.

## 1 INTRODUCTION

Ensuring safe and high‐quality treatment is of paramount concern in radiation therapy because the use of ionizing radiation is inherently associated with significant risk to both patients and staff. Albeit rare, critical radiation therapy incidents that arose due to accumulated failures of equipment and/or staff over the last two decades are well documented.^1^, ^2^ Incidents such as these, and those that occurred in other healthcare domains, have motivated healthcare providers to pursue incident mitigation strategies.

Incident learning, defined as an organization's ability to identify, report and investigate incidents, and to take corrective actions that improve the patient care system and reduce the risk of recurrence,^3^ is one such incident mitigation strategy. A key component of radiation therapy incident learning is a voluntary incident reporting and learning system (ILS) that facilitates capture of actual incidents that affected one or more patients, as well as near‐miss events that can aid in preventing the occurrence of more severe incidents.^4^

Several groups have published recommendations for, or their method of, developing radiation therapy‐specific ILSes.^5^, ^6^, ^7^ Additionally, collaborative initiatives to harmonize radiation therapy incident reporting and learning on national and international scales are ongoing. The international Radiation Oncology Safety Information System (ROSIS) was the first of these, and findings pertaining to the initial 1074 incidents reported across 101 centers over 5 years were published in 2010.^8^

More recently in North America, two national radiation therapy reporting and learning systems have been developed and deployed with significant stakeholder participation. The Radiation Oncology Incident Learning System (RO‐ILS), which is only available for participants in the US, was launched in 2014 following a beta test that began in 2013. RO‐ILS is backed by the American Association of Physicists in Medicine (AAPM) and the American Society for Radiation Oncology (ASTRO).^9^

In Canada, the National System for Incident Reporting – Radiation Treatment (NSIR‐RT) was developed in 2014 and 2015 by the Canadian Partnership for Quality Radiotherapy (CPQR) and the Canadian Institute for Health Information (CIHI).^10^ NSIR‐RT comprises a national registry for radiation therapy incident data as well as an online interface for submitting incidents and visualizing data. The NSIR‐RT taxonomy, which is detailed in the NSIR‐RT Minimum Data Set,^11^ was developed with pan‐Canadian participation including input from all core professions involved in radiation therapy. A pilot deployment of NSIR‐RT began in September 2015 and concluded in December 2016.^10^ The results of the pilot project are currently being compiled for publication.

These broadly reaching collaborative ILSes provide channels for dissemination, discussion, and analysis of incident data among participants, and aim to improve and standardize quality of care across participating institutions.^5^, ^10^ However, the software solutions that accompany these national or international systems are not optimized for integration into the local clinical workflows of individual radiation therapy centers. Also, for confidentiality reasons, they do not capture salient details like patient and physician identifiers, which are important for local follow‐up investigations and learning.

This article describes the development and implementation of an open‐source ILS software called the Safety and Incident Learning System (SaILS)^12^, ^13^ as part of a quality and safety improvement project in our radiation therapy center. SaILS was redesigned to be fully compatible with both NSIR‐RT and the incident learning workflow in our center. Also described are key features that we added to SaILS, informed by staff feedback, in the months following its deployment. The objectives of the quality and safety project were (a) to provide improved structure to, and staff engagement with, incident learning in our center, and (b) to provide feedback to the Canadian radiation therapy community regarding the use and usefulness of NSIR‐RT. To our knowledge, SaILS is the first in‐house ILS that includes all data elements of a national or international radiation therapy ILS and simultaneously satisfies local reporting requirements.

## 2 MATERIALS AND METHODS

### 2.A Setting

Our radiation therapy center operates seven linear accelerators, including one robotic radiosurgery unit, as well as three CT simulators and one MR simulator. The patient and treatment electronic medical record (EMR) is contained in the ARIA^®^ oncology information system (Version 11.0, Varian Medical Systems, Inc., Palo Alto, CA, USA). There are 43 radiation therapy technologists (RTTs) (including the chief RTT, assistant chief RTT, and four senior RTTs), 16 medical physicists, 14 radiation oncologists, 10 dosimetrists, nine clerical staff, and five radiation oncology nurses employed in our center.

### 2.B Needs assessment

**Figure 1:**
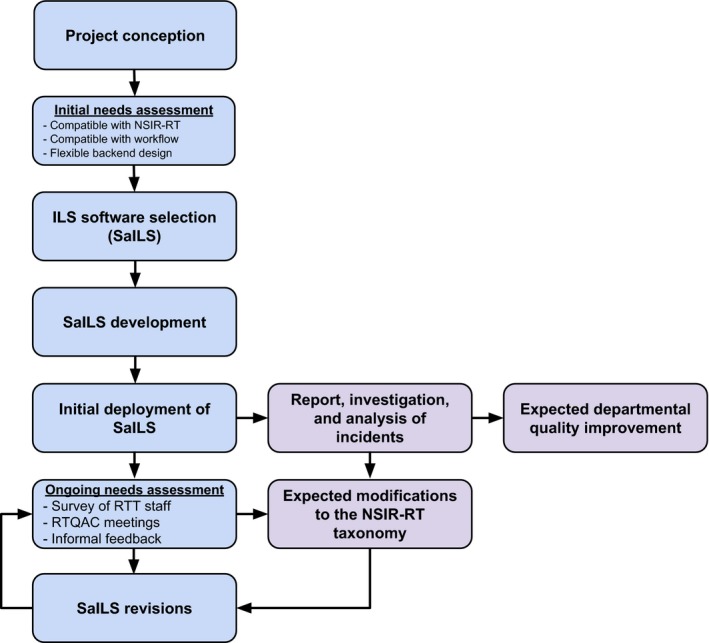
Schematic overview of the conception, development, and implementation process of our NSIR‐RT compatible ILS. Blue components depict the approach taken to determine the requirements of the ILS, as well as the iterative development of the ILS itself. Purple components represent additional aspects of incorporating the ILS into our center, but detailed discussion of which is beyond the scope of this article.

An overview of our quality and safety project is presented in Fig. 1. To meet the goals of the project, it was necessary to utilize an ILS that (a) encourages enactment of tangible change as informed by reported incidents and (b) facilitates internal and external dissemination of lessons learned. The need to use a standardized nomenclature within this ILS was thus evident, and the NSIR‐RT taxonomy was selected.

Our multiprofessional Radiation Therapy Quality Assurance Committee (RTQAC) met with representatives of the CPQR in April 2015, prior to pilot launch of NSIR‐RT. Three potential methods to incorporate the NSIR‐RT taxonomy into our center were identified:

1. Use the online interface of NSIR‐RT, hosted by CIHI, to replace our existing nonstandardized ILS.
2. Continue using our existing nonstandardized ILS and translate and transcribe incident data into the online interface of NSIR‐RT.
3. Develop a new standalone ILS that utilizes the NSIR‐RT taxonomy and is also tailored for our workflow.

The third option was selected despite requiring the largest up‐front time investment because it offered a novel platform for frontline staff to engage with the NSIR‐RT taxonomy and it solved problems that were inherent to the other two options. As discussed previously, the NSIR‐RT system (option 1) does not adequately capture patient and staff identifiers for the purposes of local incident management and was thus unsuitable for our needs. Maintaining two ILSes with distinct taxonomies (option 2) would require extra time and interpretation to translate data from one system to the other, and was thus also unsuitable. We did identify that our new ILS must offer a flexible backend because modifications to the NSIR‐RT taxonomy were anticipated over time.

Design considerations for the new ILS were informed by a review of the literature. For example, several groups have published evidence that frontline staff felt negatively about their organization's ability to learn from incidents; proportionally more‐so than management.^3^, ^14^, ^15^ This was attributed to inadequate response to incident reports, lack of feedback regarding the impact of incident reports, or both. Thus, we were motivated to incorporate tools for documented and meaningful incident follow‐up within the ILS and to offer multiple avenues of feedback (e.g., incident tracking, data visualization, etc.) to frontline staff.

Another fundamental design consideration of the new ILS was its ease‐of‐use and capability to facilitate rapid data entry. Previous studies have demonstrated that ILSes with poorly designed user interfaces (UIs) and limited features can hinder incident learning due to the significant time and frustration involved with using them to report and investigate incidents.^14^, ^15^, ^16^, ^17^ Thus, we focused initial ILS development on crafting an elegant UI with complete and user‐friendly core features. Following initial deployment of the ILS, we anticipated an ongoing needs assessment whereby staff would provide feedback and request additional features as the need was identified. The ILS would be modified accordingly, thus explicitly addressing the needs of the user‐base, demonstrating receptiveness to feedback, and encouraging continued participation.

A summary of the needs assessment is presented in Table 1.

**Table 1:** Summary of the needs assessment for our new ILS

Need	Fulfillment of Need
1) Focus on quality and safety improvements	Use an ILS that facilitates tangible and documented follow‐up to incidents
2) Focus on dissemination of lessons learned	Use a standardized nomenclature, the NSIR‐RT taxonomy, in the ILS (no double classification required)
3) Encourage ongoing participation of frontline staff	Use an ILS with a user‐friendly design Offer multiple types of feedback to frontline staff Modify the ILS as informed by staff feedback
4) Capability to accommodate future modifications to NSIR‐RT	Use an ILS with a flexible backend
5) Efficient upload of data to the NSIR‐RT registry	Use an ILS that facilitates direct upload to the NSIR‐RT registry (no double entry required)

### 2.C Software selection

Although numerous commercial solutions with robust features were identified, none already included the NSIR‐RT taxonomy nor the facility to easily incorporate a new taxonomy. Thus, a custom solution was required. The open‐source Safety and Incident Learning System (SaILS), was selected as a starting point for development. SaILS offered a well‐designed UI and facilitated a workflow similar to that envisioned by our RTQAC. Since it was open‐source it was reconfigurable to satisfy our needs.

### 2.D Development, deployment, and evaluation

We redeveloped SaILS in accordance with our needs assessment to allow compatibility with NSIR‐RT and flexibility to accommodate future changes to the taxonomy. The software was developed by a medical physics graduate student with a background in physics and computer science. The initial development period of SaILS lasted approximately 4 months and included a complete restructuring of the software backend and database. Use of the NSIR‐RT taxonomy expedited the initial development period because we did not have to create our own taxonomy from scratch.

We identified four core components of our new version of SaILS, each of which was developed from scratch or significantly modified from the original version. These components are:

1. An incident reporting interface (modified for NSIR‐RT compatibility) – Provide a blank incident report form that may be filled and submitted by users.
2. An incident investigation interface (modified for NSIR‐RT compatibility) – Provide incident investigation forms to be used by investigators to elucidate additional details on reported incidents and facilitate various outcomes.
3. Incident tracking functionality (developed from scratch) – Allow users to search for an incident and view a unique summary page that highlights key details from the report and investigation.
4. A data visualization interface (developed from scratch) – Provide a form for users to define plotting parameters and generate aggregate incident distributions and trend plots.

SaILS was deployed in our center in January 2016 and subsequent feedback from all staff was encouraged at all times. Structured feedback was also obtained via a survey of RTTs in July 2016; the complete results of which are provided in supplementary Fig. S1. Both SaILS and our workflow were revised throughout the year to improve usability and staff engagement, as informed by the feedback provided. Usability was evaluated qualitatively through discussions with staff, and staff engagement was evaluated by examining aggregate incident data that were submitted.

For the first few months after deployment, the full‐time commitment of the graduate student was required to implement the necessary software revisions. Since then, SaILS has been self‐sustaining and only requires software development time as the need for new features is identified.

## 3 RESULTS AND DISCUSSION

### 3.A Initial deployment

#### 3.A.1 Workflow redesign

**Figure 2:**
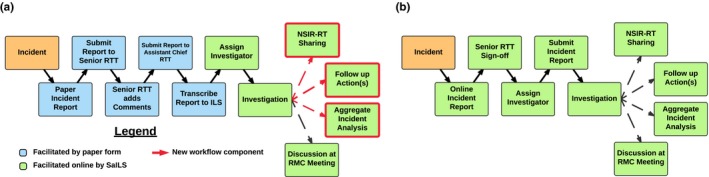
The radiation therapy incident learning workflow in our center. Elements connected by dashed lines correspond to possible outcomes of an investigation. (a) Workflow that accompanied the initial deployment of SaILS (Jan. 2016–Oct. 2016). (b) Workflow after switching to fully online incident reporting (Nov. 2016–present).

The original version of SaILS was reconfigured to facilitate the existing incident learning workflow in our clinic with only minor workflow adjustments, as shown in Fig. 2(a). The fundamental components of the previous workflow included an initial paper incident report, follow‐up investigation, and if necessary, discussion at an RTQAC meeting. Use of paper forms was motivated by senior RTTs who identified the handoff of a report from an RTT to a senior RTT as facilitating meaningful discussion that might otherwise be lost. This workflow also ensured that the assistant chief RTT was kept abreast of incidents reported by RTTs because he transcribed all paper reports into our electronic system. The new workflow deployed alongside SaILS was similar to our previous workflow but utilized new paper forms with unique incident ID numbers and tear‐off receipts to allow staff to follow‐up on their incident reports online. Additionally, compared to our previous ILS, SaILS could better facilitate multiple possible outcomes associated with incident investigations as indicated in red in Fig. 2(a).

In the redesigned workflow, follow‐up investigations allowed investigators to elucidate additional details of reported incidents and facilitate various outcomes as appropriate. Eligible investigators included all clinical medical physicists, the chief radiation oncologist, the head nurse, the chief RTT, the assistant chief RTT, and all senior RTTs. Possible outcomes of an investigation included (a) discussion of the incident at an RTQAC meeting, (b) follow‐up actions, (c) internal dissemination of lessons learned, and (d) sharing the incident data with the national NSIR‐RT registry.

#### 3.A.2 Software design ‐ Backend

The NSIR‐RT compatible version of SaILS is open‐source and is publicly available for download as a Git repository on Bitbucket: https://bitbucket.org/mcgillmedphys/sails_nsir. The software is quite portable and is thus readily usable by an institution that uses or wants to use NSIR‐RT. Installation instructions are provided in the repository and require only a basic understanding of Python and UNIX‐style command prompts. SaILS was developed using the Django Python web framework (Version 1.6.11, Django Software Foundation) and incorporates a MySQL database (Version 14.14, Oracle Corporation) on the backend. Django employs a model‐view‐template design philosophy wherein:

1. Models are Python classes that are an abstraction of the backend database.
2. Views are Python classes that define the data to be displayed and collected on each webpage.
3. Templates are dynamic HTML files that define a webpage layout in accordance with a corresponding view.

**Figure 3:**
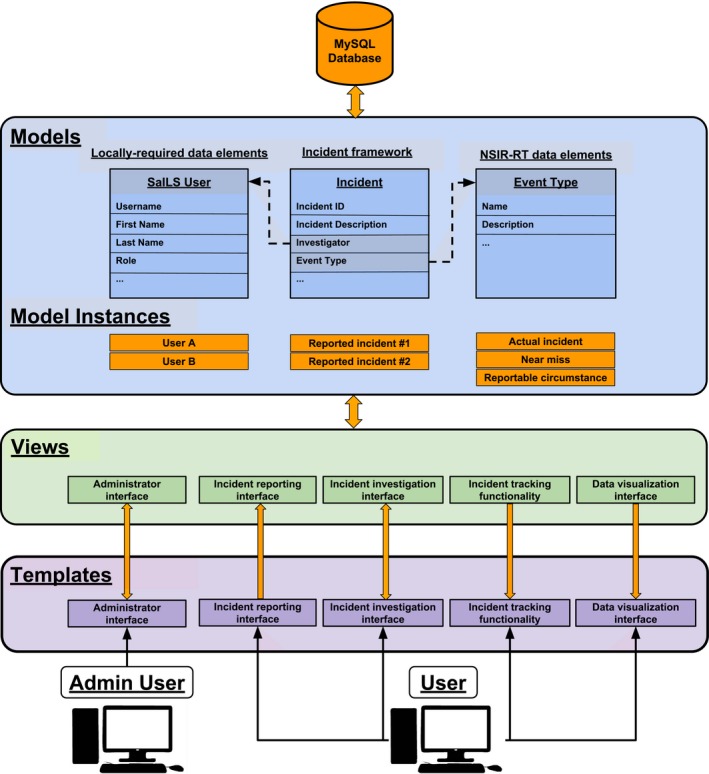
Schematic diagram of the Django model‐view‐template design philosophy as applied to SaILS. The incident model provides a framework for classifying and storing incidents. It includes fields for every NSIR‐RT data element, for example the Event Type, and every locally‐required data element, for example the Investigator. Models for all select‐type data elements were created, the instances of which correspond to coded values for that data element as defined in the NSIR‐RT MDS (e.g., Actual incident is a coded value of the Event Type data element).^11^ Views, representing the four core components and the administrator interface of SaILS, define the data (i.e., model instances) to be displayed to the user and handle saving new data provided by the user. Corresponding to each view is a template that defines the webpage layout with which the user will interact. Data and the transfer of data between the database and the user are shown in orange.

A schematic overview of the model‐view‐template design philosophy as applied to SaILS is presented and discussed in Fig. 3. The user‐friendly administrator interface of SaILS allows frontend modification of model instances (e.g., reported incidents, user profiles, possible event types, etc.). The model‐view‐template design and accompanying administrator interface were used to allow for future revisions of the NSIR‐RT taxonomy to be easily incorporated into SaILS. Also, this robust backend design should allow other users to implement their own taxonomy within SaILS with only a moderate development effort required.

#### 3.A.3 Software design ‐ Frontend

**Figure 4:**
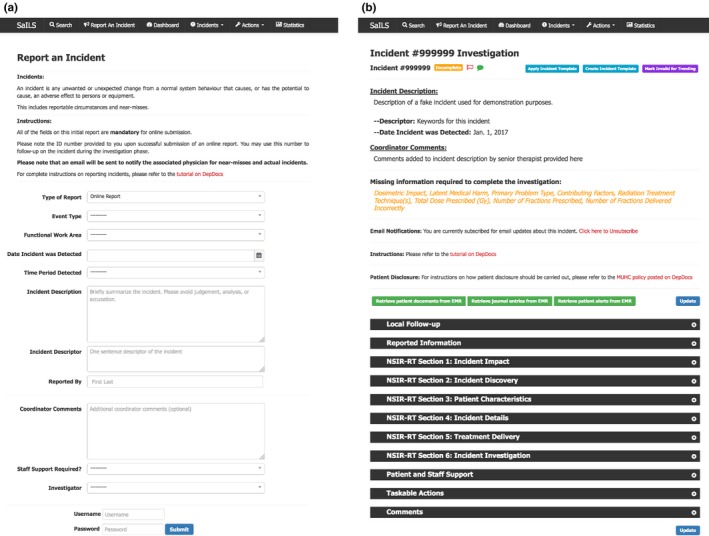
Screenshots of the incident reporting and investigation interfaces of SaILS. (a) Incident reporting interface: Instructions are provided at the top of the page, and the data elements to be reported are provided underneath. Additional data elements appear dynamically as required. For example, if the Event Type is set as Actual Incident or Near‐miss, then patient‐specific data elements like Patient ID must be included in the report. (b) Incident investigation interface: Key information is provided at the top of the page, including a list of mandatory data elements (in orange). The black header bars can be clicked to toggle display of the data elements included in the corresponding section. All data elements are displayed by default when an investigation page is loaded.

**Figure 5:**
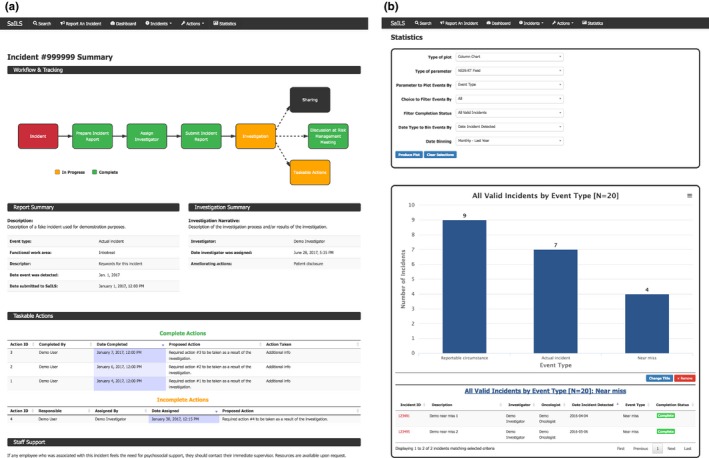
Screenshots of the incident tracking functionality and data visualization interface of SaILS. (a) Example incident summary: A dynamic flowchart depicting the current status of the incident investigation is included at the top of the page. Relevant report and investigation details are provided in tables below the flowchart. All taskable actions (a user‐requested feature) associated with the incident are listed in additional tables underneath. (b) Data visualization interface: A form is provided at the top of the page that allows users to specify plotting parameters. An example column chart of incidents distributed by the Event Type data element is shown. The list of near miss incidents underneath the plot was generated by clicking the near miss column; a feature which was requested by users.

Screenshots showing the frontend web pages of the four core components of SaILS are provided in Figs. 4 and 5.

##### Incident reporting interface

The incident reporting interface is shown in Fig. 4(a). This interface was present in the original version of SaILS but was revised to facilitate incident reports that are compatible with NSIR‐RT. Only a limited number of data elements, including a text description of the incident, are required in the initial report to minimize the time required to fill out reports. An investigator must be assigned before a report can be submitted to facilitate timely completion of incident investigations. The investigator is notified via email when they are assigned an investigation.

We opted not to allow anonymous incident reporting based on recommendations found in the literature.^5^, ^6^ Although Ford et al. did recommend allowance of anonymous reporting, they articulated that frequent anonymous reports may indicate that staff perceive the incident learning culture as punitive. By extension, we believe that simply presenting users with an anonymous reporting option may allude to a punitive aspect of SaILS. Also, anonymous reports may impede an investigator's ability to complete their investigation if they require additional details about the incident but do not know with whom to inquire. The decision was thus made to not allow anonymous reporting but to ensure that normative information submitted to SaILS is confidential and used in a nonpunitive manner.

##### Incident investigation interface

An investigation interface was also present in the original version of SaILS. The revised interface is shown in Fig. 4(b). Incident investigations are only readable by investigators to provide confidentiality, and are only editable by the assigned investigator. All NSIR‐RT data elements and locally required data elements are included in the interface and the investigator is encouraged to provide values for each. However, the investigator is not permitted to change information that was provided in the initial incident report. A notable data element that we added to the investigation interface is a mandatory text field named Investigation Findings and Actions Taken that allows investigators to articulate the investigation process and associated outcomes. It complements the Incident Description (from NSIR‐RT) provided by the individual who reported the incident and archives a clear narrative that may be difficult to interpret otherwise.

Investigations are automatically completed once values for all mandatory data elements, including those defined as such in the NSIR‐RT taxonomy,^11^ have been provided. However, the investigator may choose to close an incident investigation early if the incident is deemed “invalid”. Such incidents are perceived as problems when they are reported, but upon review by the investigator are found to be nonproblematic. Any user who can view an investigation can flag it for discussion at the next RTQAC meeting. The investigator may also choose to reassign the investigation to somebody else as necessary.

##### Incident tracking functionality

Incident tracking is facilitated via a robust search feature and incident summary pages that are generated for each incident. An example summary page is shown in Fig. 5(a). Users may search for incidents by incident ID, patient ID, or keyword and are provided with links to the summary pages of all matching incidents. A summary page, which is visible to all staff, includes a diagram that indicates the status of the investigation and lists key information provided in the incident report and investigation. No information about the patient or staff who reported the incident is provided on an incident summary page.

##### Data visualization interface

The data visualization interface, shown in Fig. 5(b), allows users to generate pie charts, column distribution charts, and column trend charts of aggregate incident data submitted to SaILS. Incidents may be sorted by the coded values of select‐type data elements and filtered by date range and investigation completion status.

### 3.B Postdeployment revisions

#### 3.B.1 Workflow revision

Twenty‐six of 37 RTTs surveyed responded to a questionnaire circulated in July 2016, yielding an overall response rate of 70%. Twenty‐three of the respondents answered a question regarding their preferences for submitting incident reports, and 18 (78%) indicated they would prefer to submit reports electronically instead of on paper. This motivated a change to the incident reporting workflow to eliminate paper forms, as depicted in Fig. 2(b). The change was implemented in October 2016 after all RTTs were provided with institutional email accounts, necessary for login to SaILS. RTTs now enter incident reports directly online using SaILS, but reports must be reviewed and signed off by a senior RTT to be submitted. Like the previous workflow, this online “handoff” facilitates discussion between the RTT and a senior RTT when an incident is reported and eliminates the inconvenience of paper forms. Also, email notifications are automatically sent to the chief and assistant chief RTT when an incident report is submitted by an RTT to ensure that management is aware of all incidents. The assistant chief RTT is suggested as the investigator for all RTT‐submitted incidents, but the investigator may be reassigned as appropriate.

#### 3.B.2 Software revisions ‐ Incident investigation interface

The four most impactful features that were added to the incident investigation interface postdeployment, based on investigator feedback, are described in further detail below.

##### Connection to EMR

Five buttons were added to the investigation interface that allow users to retrieve patient‐specific information and documentation directly from our EMR. For example, the “Retrieve treatment info from EMR” button will auto‐fill the Radiation Treatment Technique, Prescribed Dose, and Number of Fractions Prescribed data elements of the NSIR‐RT taxonomy. The remaining buttons auto‐fill patient specific information, and display documentation that may aid in the investigation. The SaILS source code includes template files to aid other users in implementing this feature alongside their EMR (minimal development effort should be required for users of Varian's ARIA^®^ oncology information system).

##### Taskable actions

Taskable actions were developed to facilitate tangible and documented follow‐up to incident investigations, and reduce dependence on verbal relay of information. They can be created by any user with investigative privileges, not just the investigator of a particular incident. Ninety‐six actions were created for 85 different incidents after the feature was launched in March 2016. In other words, actions were generated for 36% of the total number of incidents that were either reported after the feature was launched or had not been completely investigated beforehand (*N* = 233). These actions consisted of staff reminders, scheduling of case debriefing sessions, scheduling of inter‐ and intradepartmental meetings, and general communication between SaILS users. All taskable actions associated with an incident are included on its incident summary page, thereby providing automatic feedback to frontline staff.

##### Investigation reminders

Automated periodic email reminders for incomplete investigations were incorporated because investigators noted that they occasionally forgot about investigations of minor severity if they were unable to complete them immediately upon assignment. It was found that investigations were frequently completed soon after reminders were sent, most notably for incidents that were incomplete before the feature was launched.

##### Incident templates

Investigators requested the ability to expedite investigation of incidents that were similar to those previously investigated. Similar incidents were typically reported either (a) before the underlying problem, identified in the initial report, had been resolved or (b) due to other factors that led to heightened awareness of that type of incident in our center. Thus, template creation was added to SaILS, which allows investigators to save values inputted into eligible select‐type data elements. Eight templates were created by investigators, most of which encompassed incidents reported in treatment planning regarding unclear planning instructions and tasking errors. The investigations for twenty‐three of the 216 incidents (11%) that were either reported after the feature was launched or had not been completely investigated beforehand, were completed using a template.

#### 3.B.3 Software revisions ‐ Data visualization interface

Functionality was added to the data visualization interface to allow users to click on any chart element (i.e., any column or pie wedge) and view the list of incidents corresponding to that element, as depicted at the bottom of Fig. 5(b). This allows users to quickly generate lists of incidents that were categorized similarly. These incident lists may be reviewed when performing aggregate incident analysis to examine trends in incident data.

#### 3.B.4 Software revisions ‐ Incident tracking functionality

RTT responses to three survey questions regarding incident tracking and feedback are provided in Table 2. The full survey and results are provided in supplementary Fig. S1. Because RTTs were, at that time, still reporting incidents via paper forms, they felt that they were not engaged with SaILS and it was cumbersome for them to seek out follow‐up information on their incident reports. This is expected to have contributed to the high proportion of “No” responses to questions four and five as indicated in Table 2. Now that RTTs have institutional email addresses, SaILS is being reconfigured to accommodate RTT user‐accounts with personalized incident lists.

**Table 2:** Three questions regarding incident tracking and feedback that were posed in our survey of RTTs (n/N = 26/37)

Question (paraphrased)	Yes	No	Answer rate among survey respondents (n_i_/n)
Q4) Have you used SaILS to follow‐up on any incidents?	16% (4)	84% (21)	96% (25/26)
Q5) If yes to the previous question, have you found the provided feedback to be useful or informative?	40% (2)	60% (3)	19% (5/26)
Q8) Would you like to receive newsletters that highlight certain anonymized incidents and corresponding actions?	100% (22)	0% (0)	85% (22/26)

The unanimous response to question eight prompted the creation of an incident learning newsletter, the first of which is included in supplementary Fig. S2. It was circulated in our center in January 2017 to summarize our first year of experience with SaILS. We aim to maintain a quarterly schedule for newsletter circulation and are developing a blog‐style news page within SaILS that will act as a newsletter archive. SaILS admin users and investigators will also be able to publish other news items, for example case studies, as appropriate.

### 3.C Staff engagement

SaILS user‐accounts for 44 staff members in our center were created in 2016, including the chief RTT, assistant chief RTT, head nurse, four senior RTTs, all medical physicists, all dosimetrists, and all radiation oncologists.

**Figure 6:**
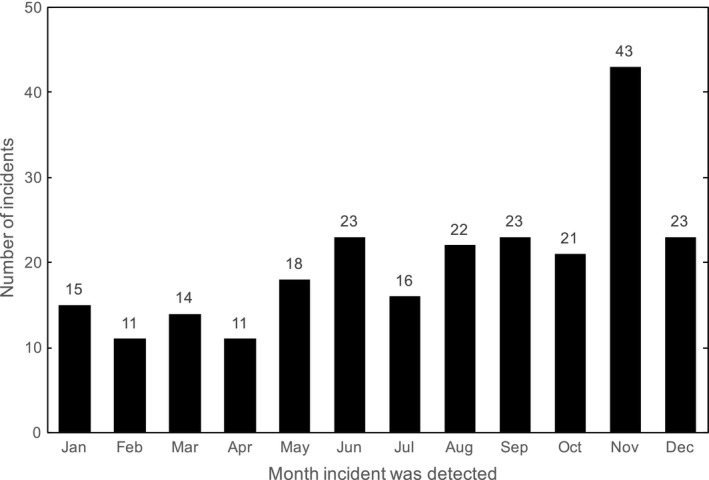
Number of valid incidents detected per month in 2016 (*N* = 240).

There were 240 valid incidents detected and reported in 2016, of which 199 are presently investigation‐complete (83%). Also, there were 38 incidents that were reported that were later deemed invalid by the assigned investigator. The distribution of valid incidents by the month they were detected is shown in Fig. 6. It was observed that the number of incidents detected per month tended to increase over the course of the year, which indicates that staff participation in the incident learning process improved following implementation of our quality and safety project. This may be partially attributed to the flexibility of SaILS and our workflow, which were refined to meet staff needs as they arose.

A significant increase in the number of incidents reported in November may be attributed to the implementation of online incident reporting for RTTs in October. In other words, the cumbersome nature of submitting paper forms may have been a deterrent to reporting incidents. Although there were fewer incidents detected and reported in December than November, the quantity was consistent with previous months and we are confident the change in workflow was beneficial to the overall incident learning process in our center. The number of incidents submitted to SaILS per month will continue to be monitored to gauge staff participation.

**Figure 7:**
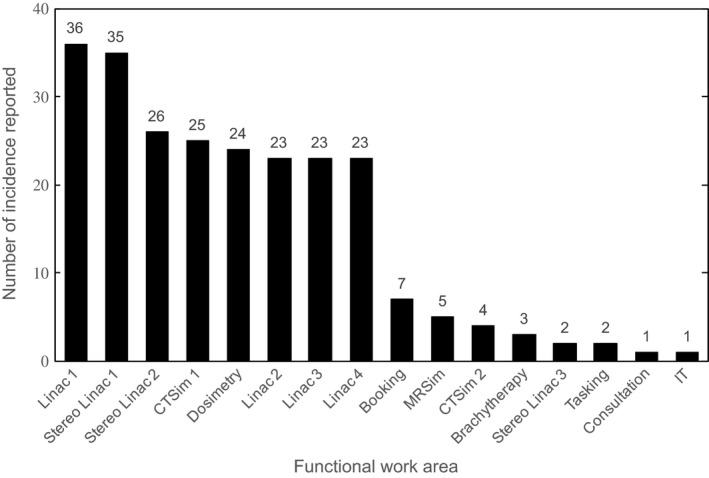
Distribution of all 240 valid incidents detected in 2016 by the functional work area from which they were reported.

Overall, a high level of RTT participation in both detecting and reporting incidents was observed. Twenty of the 25 respondents (80%) who answered question two in our survey of RTTs indicated they had submitted an incident report since SaILS was deployed. This percentage is likely to have increased since the survey was circulated in July 2016. Also, a significant number of incidents were reported from each treatment unit and imaging suite as shown in Fig. 7. The majority of reported incidents were detected by RTTs as shown in Table 3, which also compares our staff engagement with published results for other ILSes.^8^, ^18^ The SaILS data provided in Table 3 correspond to values inputted into the (optional) Health Care Provider(s) and/or Other Individual(s) Who Detected the Incident data element of the NSIR‐RT taxonomy.^11^ NSIR‐RT data were used with permission from CIHI and include all incidents submitted prior to May 31st, 2017. We aim to maintain a high‐level of RTT participation by continuing to provide feedback to staff that demonstrates the importance of incident reports.

**Table 3:** Distribution of reported incidents by the role of the individual who detected the incident for SaILS and other collaborative incident learning systems

Role	SaILS	ROSIS^8^	RO‐ILS^18^	NSIR‐RT
Radiation therapy technologist	57% (137)	61%	23%	73%
Treatment planner or dosimetrist	8% (20)	4%	4%	3%
Radiation oncologist	1% (2)	8%	4%	2%
Medical physicist	<1% (1)	9%	13%	2%
Other	<1% (1)	3%	4%	2%
Unspecified/Unknown	33% (79)	15%	53%	21%

As indicated in Table 3, a large percentage of incidents that were reported to other ILSes were also detected by RTTs. However, in our center, the medical physics team was attributed to the detection of a single incident that was reported using SaILS during 2016. This result is notably lower than equivalent results in ROSIS and RO‐ILS, but appears consistent with Canadian practice (acknowledging that 6% of incidents submitted to the NSIR‐RT pilot study were from our center). We suspect there is a high threshold for physicist incident reporting in our center and that near‐miss incidents detected by physicists, particularly in treatment planning, may be under‐reported. This serves as motivation to better engage our physics team and encourage their participation with SaILS so as to better document and disseminate lessons learned from near‐misses. The percentages of reported incidents that were detected by dosimetrists and radiation oncologists are, however, relatively consistent with other ILSes.

## 4 DISCUSSION OF THE ROLE OF NSIR‐RT

Collaborative incident learning in radiation therapy, including use of a shared nomenclature, has the potential to improve and standardize quality of care across participating institutions.^5^, ^10^ Use of a shared nomenclature and anonymized, aggregated incident data allowed us to objectively compare staff participation in our incident learning process with other institutions. We believe this may powerfully scale to other types of quality benchmarking across institutions. This will allow participating institutions to set attainable goals for quality improvement and, as proposed in the literature, may standardize their quality of care.

Additionally, use of the NSIR‐RT taxonomy has improved the structure of incident learning within our center. The extensive use of data elements with coded values facilitated grouping of incidents with similar characteristics for aggregate incident analysis. Concerns were initially raised by investigators regarding the time‐consuming nature of classifying incidents within the NSIR‐RT taxonomy, particularly when the required data existed elsewhere. However, these concerns were alleviated by incorporating a direct connection to our EMR and by providing incident templates within the investigation interface, which minimized redundant data entry.

Placement of the NSIR‐RT taxonomy in the hands of frontline staff and investigators in our center provided us with a unique platform for evaluation of the national taxonomy. A detailed summary of the recommendations that were informed through use of SaILS will be the subject of a forthcoming article. However, it is important to note here that we recommend making certain data elements mandatory for all incident reports, such as the Health Care Provider(s) and/or Other Individual(s) Who Detected the Incident data element. The certainty of the comparative results discussed earlier would be improved if a value for this had been specified for all incidents where it was determinable. Capturing data elements such as this one should not require significant additional time investment and an “Unknown” option is available in the taxonomy for cases where it is truly unknown.

Use of the NSIR‐RT taxonomy allowed us to deploy SaILS much sooner than if we had attempted to create a taxonomy from scratch. Also, the NSIR‐RT taxonomy was constructed using a modified Delphi method to achieve consensus among a multi‐professional group of experts from across Canada.^10^ While not universally true, an internally developed taxonomy is unlikely to be as intuitive or comprehensive as that of NSIR‐RT. This was demonstrated by our previous ILS and its inability to be sustained. Participation in a multi‐institutional initiative, such as NSIR‐RT, also includes benefits such as:

1. Broad scrutiny of the taxonomy, rapid identification of its deficiencies, and ultimately its continuous improvement.
2. Access to shared educational opportunities (e.g., webinars) that promote consistency in incident categorization both internally and nationally.
3. Use of a shared nomenclature and infrastructure for easier multi‐institutional dissemination of lessons learned.

The capability of SaILS to quickly be adapted to modifications to the NSIR‐RT taxonomy was tested in August 2016 after the first NSIR‐RT Update newsletter was circulated. Among other things, the newsletter described several minor adjustments to existing data elements in the taxonomy. These adjustments were easily incorporated into SaILS owing to the flexible backend design described in Section 3.A.2. We are thus optimistic about the feasibility of incorporating additional changes in the future as needed.

Currently, incident data must be manually transcribed from SaILS into the national registry. The transcription process takes approximately three minutes for an incident that has been fully classified. Although this is faster than translating and transcribing incidents classified using a different taxonomy into NSIR‐RT, the process is still cumbersome. To eliminate this burden, we are developing a batch upload feature that allows direct upload of incident data from SaILS to NSIR‐RT in collaboration with CIHI. This feature will meet the final need in our needs assessment (Need #5 in Table 1), thereby yielding an NSIR‐RT‐ and clinically compatible software that eliminates double data entry.

## 5 CONCLUSIONS

We have redesigned the open‐source Safety and Incident Learning System (SaILS) to be completely compatible with the Canadian NSIR‐RT taxonomy and to facilitate our center's incident learning workflow. Use of SaILS has improved the structure of incident learning in our center by facilitating documentation of incidents and follow‐up. Staff engagement with SaILS was improved following its deployment by adapting it to address staff needs as they arose and by providing feedback on reported incidents. Although it is unlikely that the NSIR‐RT taxonomy was necessary to achieve the level of engagement we attained with SaILS, its use facilitated faster software deployment, provided improved structure to incident classification, and allowed for shared educational opportunities.

Use of an ILS such as SaILS that facilitates local incident learning and dissemination at the national level will, we believe, benefit the radiation therapy community and the patients we serve.

## CONFLICT OF INTEREST

No conflicts of interest.

## Supporting information

Figure. S1. (a) Summary of an incident reporting and learning survey of RTTs in our radiation therapy center (questions 1‐4). (b) Summary of an incident reporting and learning survey of RTTs in our radiation therapy center (questions 5‐8).
Figure. S2. (a) Preliminary incident reporting and learning newsletter circulated in our radiation therapy center (front). (b) Preliminary incident reporting and learning newsletter circulated in our radiation therapy center (back).
