# Supplementary material for: Development and implementation of a radiation therapy incident learning system compatible with local workflow and a national taxonomy
Source: J Appl Clin Med Phys. 2017 Nov 22;19(1):259–70. doi: 10.1002/acm2.12218 (PMC5767999; doi:10.1002/acm2.12218)
Supplement: Supplementary file 1 — Figure. S1. (a) Summary of an incident reporting and learning survey of RTTs in our radiation therapy center (questions 1‐4). (b) Summary of an incident reporting and learning survey of RTTs in our radiation therapy center (questions 5‐8). Figure. S2. (a) Preliminary incident reporting and learning newsletter circulated in our radiation therapy center (front). (b) Preliminary incident reporting and learning newsletter circulated in our radiation therapy center (back). [file ACM2-19-259-s001.pdf]

Supplementary Material

Are you aware of the incident reporting system in our radiotherapy department?

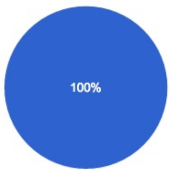

Yes	**26**	100%
No	**0**	0%

Have you submitted an incident report within the last 6 months?

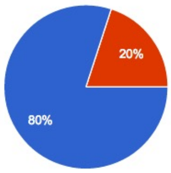

Yes	**20**	80%
No	**5**	20%

The current electronic incident reporting system (SaILS), as well as revised paper incident report forms, went live in January 2016. Do you prefer this new system or the previous system?

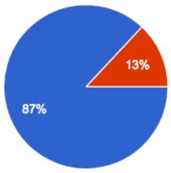

New system	**20**	87%
Old system	**3**	13%

Have you used the new electronic incident reporting system to follow-up on any of your reported incidents?

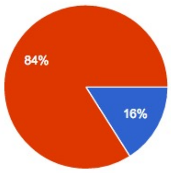

Yes	**4**	16%
No	**21**	84%

Fig. S1. (a) Summary of an incident reporting and learning survey of RTTs in our radiation therapy centre (questions 1-4).

If you answered yes to the previous question, have you found the feedback provided to be useful/informative?

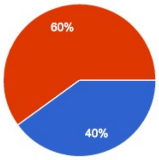

Yes	**2**	40%
No	**3**	60%

On a scale of 1 to 10, how well do you believe our radiotherapy centre is willing and able to learn from previous incidents and make positive system changes when necessary?

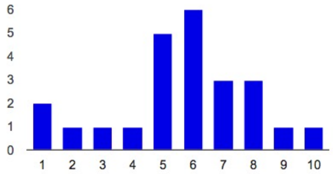

Unable to Learn from Previous Incidents: 1	**2**	8.3%
2	**1**	4.2%
3	**1**	4.2%
4	**1**	4.2%
5	**5**	20.8%
6	**6**	25%
7	**3**	12.5%
8	**3**	12.5%
9	**1**	4.2%
Demonstrated Ability to Learn from Previous Incidents: 10	**1**	4.2%

Would you prefer to continue submitting paper incident reports, or would you rather submit incident reports electronically via a web page?

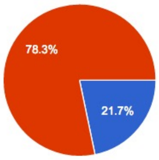

Paper incident reports	**5**	21.7%
Electronic incident reports via a web page	**18**	78.3%

Would you like to receive periodic (quarterly or biannually) newsletters that highlight particular anonymized incidents as well as actions which have been taken to prevent their recurrence?

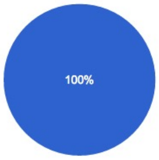

Yes	**22**	100%
No	**0**	0%

Fig. S1. (b) Summary of an incident reporting and learning survey of RTTs in our radiation therapy centre (questions 5-8).

2016 Incident Learning: Year in Review

Radiation Oncology Incident Learning Update | Issue #1 | Jan. 2017

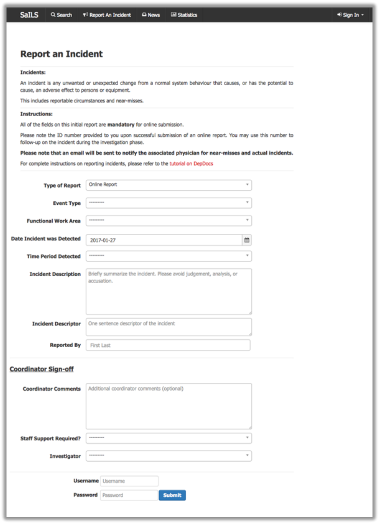

About SaILS

- SaILS is the incident reporting and learning system in the Departments of Radiation Oncology and Medical Physics
- The goal of using SaILS is to aid in identifying and solving system-level patient safety issues in our departments
- SaILS may be accessed via http://medphys/
- SaILS uses the taxonomy of the Canadian National System for Incident Reporting – Radiation Treatment (NSIR-RT) to standardize incident classification across Canada
- Learn more about NSIR-RT at http://www.cpqr.ca/programs/national-incidents-reporting/

Event Type classification

Use this flowchart to properly classify the Event Type of an incident!

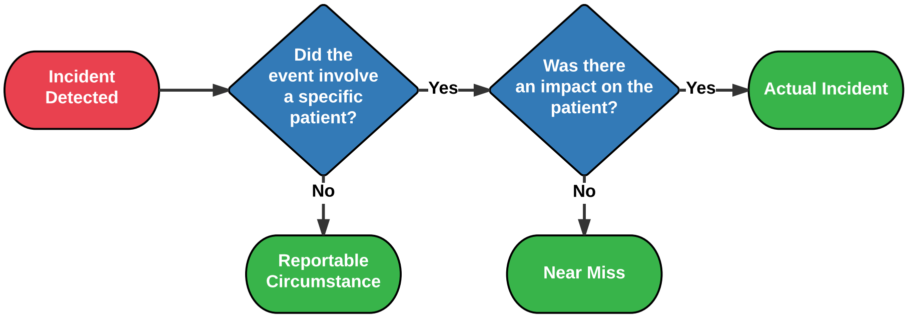

Fig. S2. (a) Preliminary incident reporting and learning newsletter circulated in our radiation therapy centre (front).

Incident Learning Update | Issue #1 | January

Incident Data

Number of incidents reported per month

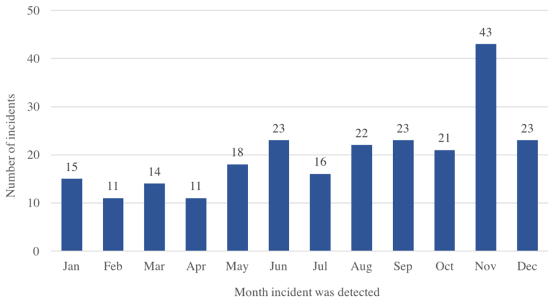

- 240 incidents reported in 2016
- More incidents reported in November after switching to online incident reporting for technologists
    - **Good!** Reduced barrier to data entry

Distribution of incidents by Event Type

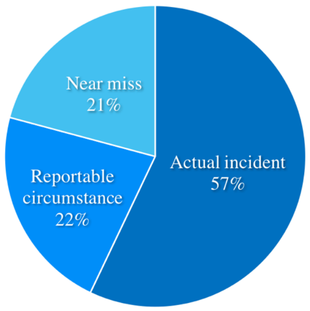

- Majority of incidents classified as actual incidents
- Higher proportion of near misses reported in November and December
    - **Good!** Reporting more incidents before reaching patients

View statistics like these and more by visiting SaILS and clicking the *Statistics* tab

Results of Staff Feedback

- Technologists were surveyed in July 2016. Outcomes of this survey include:
- This newsletter!
    - 100% of respondents requested periodic newsletters
- Online incident reporting for technologists
    - 78% of respondents requested that reporting be done online instead of on paper forms
    - Result: faster turnaround time on investigations

Coming Soon!

- Additional newsletters (one per quarter)
- SaILS user accounts for all technologists
    - View personalized list of submitted incidents
    - Examine incident summaries to review actions that were taken as a result of an incident
- Case studies
- Another survey RE: incident reporting practices & feedback

Fig. S2. (b) Preliminary incident reporting and learning newsletter circulated in our radiation therapy centre (back).
